# Editorial: Physiology and Pathophysiology of the Extracellular Calcium-Sensing Receptor

**DOI:** 10.3389/fphys.2018.00413

**Published:** 2018-05-08

**Authors:** Enikö Kallay

**Affiliations:** Department of Pathophysiology and Allergy Research, Medical University of Vienna, Vienna, Austria

**Keywords:** calcium-sensing receptor, physiology, structure-activity relationship, mutations, G protein-coupled receptor

Calcium is a universal signal carrier for biological information, being one of the most specific and most selective messengers in nature. It is involved in multiple signaling cascades - critical for cell survival, differentiation, or death. Within the cell, calcium controls several different signaling pathways, including those that regulate cell growth and cell death. The extracellular calcium-sensing receptor (CaSR) is the fundamental tool used by cells to detect subtle changes in extracellular calcium (Ca^2+^) and has been the subject of intense research activity since it was first cloned 25 years ago (Brown et al., 1993).

The class C G protein-coupled receptor CaSR plays a pivotal role in systemic calcium homeostasis by regulating parathyroid hormone (PTH) secretion and urinary calcium excretion. There is emerging evidence that the CaSR represents a key molecule in physiology, as it controls such diverse processes as hormone secretion, ion channel activity, gene expression, modulation of inflammation, proliferation, differentiation, and apoptosis, depending on cell type.

Initially, the studies on the CaSR were focusing on calcitropic tissues with obvious roles in calcium homeostasis such as parathyroid, kidney, and bone. By now it has become clear that the receptor is expressed in many other tissues, without any obvious role in this process, such as neurons, lung, skin, placenta, breast, endothelium. Abnormal CaSR expression and function is implicated not only in calcitropic disorders such as hyper- and hypoparathyroidism, but also in diseases linked to non-calcitropic systems, such as the nervous, reproductive, and respiratory system, and even in diseases such as chronic inflammation and cancer (Brennan et al., 2013).

The CaSR binds different ligands and interacts with multiple heterotrimeric G protein subtypes thereby regulating highly divergent downstream signaling pathways, depending on the cellular context. A broad understanding of the genetic, molecular, and cellular regulation of CaSR expression and signaling is crucial to comprehend both its importance in normal physiology and to devise unique, targeted drugs to treat diseases linked to impaired CaSR expression or function.

Since 1993, when the CaSR was first cloned and characterized, more than 3,600 citations are listed in PubMed under the search term “calcium-sensing receptor.” The aim of this research topic is to group research articles and reviews that highlight the latest discoveries on the multifaceted roles of the CaSR both in health and disease.

The excellent overview of Conigrave describes how CaSR regulates parathyroid function in health and disease and how studies of this receptor have led to finding new approaches to treat various disorders of parathyroid function and calcium metabolism. He provides an account of the first characterization of the pivotal parathyroid Ca^2+^ sensing mechanism and how key biochemical features of the signaling mechanisms were exploited to clone the CaSR.

In order to understand the function of the CaSR it is important to know its structure and the factors responsible for its regulation. The crystal structure of the extracellular domain (ECD) of the CaSR has been deciphered only recently (Geng et al., 2016; Zhang et al., 2016). In the present research topic Zhang et al. review the newest insights into the molecular basis of the structure and functional cooperativity of the CaSR, describing the structure–function relationships of the various domains of the CaSR protein. Numerous mutations of the *CASR* gene leading to human diseases were identified. The paper from Zhang et al. summarizes the four major types of mutations that lead to calcitropic diseases, a subject that is considered in more detail by Roszko et al. In their paper Roszko et al. describe two forms of hypoparathyroidism: autosomal dominant hypocalcaemia (ADH) type 1, caused by activating mutations of the *CASR* gene and ADH type 2, caused by gain-of-function mutations in the alpha subunit of the G protein G11 (Gα11), a key signaling partner of the CaSR.

The level of the CaSR mRNA and protein is very different in the different tissues. Hendy and Canaff reviewed the efforts made by numerous groups to unveil the mechanisms that regulate CaSR expression. The multiple promoters offer the potential for tissue-specific regulated expression from one promoter vs. ligand-specific responsiveness from the other. This up-to-date review of the structure and regulation of the CaSR describes that besides epigenetic mechanisms, increased levels of different pro-inflammatory cytokines, and active vitamin D_3_, calcitriol modulate the expression of the CaSR. In the contribution of Aggarwal and Kállay the link between the CaSR and vitamin D is further analyzed, showing a multifactorial cross-talk between the two systems and demonstrating synergism in regulating multiple pathways involved in carcinogenesis. As Bikle et al. further show, mice lacking both the CaSR and the vitamin D receptor (VDR) in their epidermis, develop tumors spontaneously if fed low calcium diet, underlining the importance of both CaSR and VDR for keratinocyte differentiation and skin cancer prevention.

Next, the group of Carmen de Torres describes the role of the CaSR for the normal development of the central and peripheral nervous system, and its involvement in neuroblastoma differentiation. They suggest that pharmacological modulation of this GPCR might be beneficial in the treatment of neuroblastoma and other developmental diseases of the nervous system. Indeed, the CaSR is a key target for ${\text{Ca}}_{\text{o}}^{2+}$ signaling in neurons, as it is so well described by Jones and Smith, who show that the CaSR regulates intrinsic excitability, synaptic transmission, and neuronal activity and it is involved in the pathogenesis of acute neurological diseases like stroke, traumatic brain injury, and epilepsy. Moreover, the CaSR seems to be involved also in the pathophysiology of Alzheimer's disease. Chiarini et al. show that in the late-onset form of the disease exogenous amyloid-β_42_ oligomers are able to bind to the CaSR on neurons and astrocites, inhibit the proteolysis of the intracellular amyloid-β_42_ oligomers, leading to their accumulation. They found that selective allosteric CaSR antagonists were able to suppress these neurotoxic effects. Tharmalingam and Hampson focus on the role of the CaSR in cell differentiation and migration, and the mechanisms underlying these processes in the developing central nervous system and in cancer, suggesting that the CaSR may serve either as a tumor suppressor or oncogene. They address recent developments that show that the CaSR interacts with integrins and that the CaSR/integrin complexes may function as a universal cell migration or homing complex.

Smith et al. review the role of the CaSR in pulmonary hypertension. They show that the CaSR is upregulated in smooth muscle cells of the pulmonary arteries of patients with pulmonary artery hypertension which leads to more robust CaSR-mediated cytosolic Ca^2+^ increase and enhanced proliferation. However, pharmacological inhibition of the CaSR prevents the progression of experimental pulmonary hypertension, suggesting that targeting the CaSR may be useful as a novel therapeutic approach.

As shown by Kim and Wysolmerski in normal breast epithelial cells the CaSR ensures adequate translocation of Ca^2+^ from the blood to the milk during lactation, while inhibiting parathyroid hormone-related protein (PTHrP) secretion. Interestingly, in breast cancer cells, CaSR activation leads to higher PTHrP secretion due to a switch in G protein coupling, from Gα_i_ to Gα_s_, thus stimulating cell proliferation and inhibiting apoptosis. In her excellent review, Ellinger provides an overview of the roles of the CaSR in the different reproductive organs, bringing evidence that the CaSR is an important regulator of the physiology of mammalian reproductive and developmental processes, pointing to the necessity to explore more in depth the role of the CaSR in male and female infertility.

The role of the CaSR as mediator of adipose tissue dysfunction was examined by Roberto Bravo-Sagua et al. who show that the CaSR is a potential regulator of white adipose tissue physiology. Tang et al. cover the diverse roles of the CaSR as a nutrient sensor in the physiology of the gastrointestinal tract, which include diverse actions from modulation of acid secretion in the stomach, amino acid-stimulated release of gut hormones in the small intestine, suppression of fluid secretion in the colon, regulation of microbiome composition to inhibitory actions on the proliferation of colonic crypt cells. An active CaSR is relevant for the regulation of intestinal barrier integrity and motility. There is evidence that the CaSR may participate in regulation of food intake, and CaSR agonists affect the enteric nervous system, and are able to restore imbalanced immune responses.

## Author contributions

The author confirms being the sole contributor of this work and approved it for publication.

### Conflict of interest statement

The author declares that the research was conducted in the absence of any commercial or financial relationships that could be construed as a potential conflict of interest.

## References

[B1] Brennan S. C. Thiem U. Roth S. Aggarwal A. Fetahu I. S. Tennakoon S.. (2013). Calcium sensing receptor signalling in physiology and cancer. Biochim. Biophys. Acta 1833, 1732–1744. 10.1016/j.bbamcr.2012.12.011 23267858

[B2] Brown E. M. Gamba G. Riccardi D. Lombardi M. Butters R. Kifor O.. (1993). Cloning and characterization of an extracellular Ca^(2+)^-sensing receptor from bovine parathyroid. Nature 366, 575–580. 10.1038/366575a0 8255296

[B3] Geng Y. Mosyak L. Kurinov I. Zuo H. Sturchler E. Cheng T. C.. (2016). Structural mechanism of ligand activation in human calcium-sensing receptor. Elife 5:e13662. 10.7554/eLife.13662 27434672 PMC4977154

[B4] Zhang C. Zhang T. Zou J. Miller C. L. Gorkhali R. Yang J.-Y.. (2016). Structural basis for regulation of human calcium-sensing receptor by magnesium ions and an unexpected tryptophan derivative co-agonist. Sci. Adv. 2:e1600241. 10.1126/sciadv.1600241 27386547 PMC4928972

